# Synthesis of Novel Pyrazole Derivatives and Their Tumor Cell Growth Inhibitory Activity

**DOI:** 10.3390/molecules24020279

**Published:** 2019-01-13

**Authors:** Ying-Jie Cui, Long-Qian Tang, Cheng-Mei Zhang, Zhao-Peng Liu

**Affiliations:** Institute of Medicinal Chemistry, Key Laboratory of Chemical Biology (Ministry of Education), School of Pharmaceutical Sciences, Shandong University, Jinan 250012, China; yingjiecui@sina.cn (Y.-J.C.); lqtang.student@sina.com (L.-Q.T.); zhangcm@sdu.edu.cn (C.-M.Z.)

**Keywords:** privileged structure, pyrazoles, tubulin inhibitors, antitumor, antitumor agents

## Abstract

To find novel antitumor agents, a series of 1*H*-benzofuro[3,2-*c*]pyrazole derivatives **4a-e** were designed and synthesized. The treatment of 6-methoxybenzofuran-3(2*H*)-one **3** with LiHMDS in anhydrous tetrahydrofuran (THF) followed by reaction with 3-substitued phenyl isothiocyanate gave the thioamide intermediates, which underwent condensation with hydrazine monohydrate in dioxane/EtOH (1:1) to provide the benzofuropyrazole derivatives **4a**–**e** as well as the unexpected pyrazole derivatives **5a**–**e**. In tumor cell growth inhibitory assay, all the benzofuropyrazole derivatives were not active against the breast tumor MCF-7 cell, only **4a** was highly active and more potent than ABT-751 against the leukemia K562 (GI_50_ = 0.26 μM) and lung tumor A549 cells (GI_50_ = 0.19 μM), while other benzofuropyrazoles showed very weak inhibitory activity. In contrast, the pyrazoles **5a-e** were in general more potent than the benzofuropyrazoles **4a**–**e**. Compound **5a** exhibited a similar tendency to that of **4a** with high potency against K562 and A549 cells but weak effects on MCF-7 cell. Both pyrazoles **5b** and **5e** exhibited high inhibitory activities against K562, MCF-7 and A549 cells. The most active compound **5b** was much more potent than ABT-751 against K562 and A549 cells with GI_50_ values of 0.021 and 0.69 μM, respectively. Moreover, **5b** was identified as a novel tubulin polymerization inhibitor with an IC_50_ of 7.30 μM.

## 1. Introduction

Privileged structures are defined as molecular frameworks that are able to provide useful ligands for multiple types of receptors or enzymes through proper structural modifications. In combination with their favorable drug-like properties, privileged structures or scaffolds are widely used in rational drug design to find new lead compounds or drug candidates [1,2,3]. Pyrazole derivatives represent one of the most active classes of compounds that possess a wide spectrum of biological activities, including antibacterial and antifungal [4,5], antitumor [6,7], anti-inflammatory and analgesic [8,9], antitubercular [10], antiviral [11,12], anti-Alzheimer’s [13,14], α-glucosidase inhibitory [15], anti-diabetic [16], antileishmanial [17,18], anti-malarial [19], radioimaging [20], acaricidal and insecticidal [21,22] activities. As a privileged scaffold, pyrazole has been recently widely used in the design of anticancer agents for a multiple of tumor targets [23]. 

**Figure 1:**
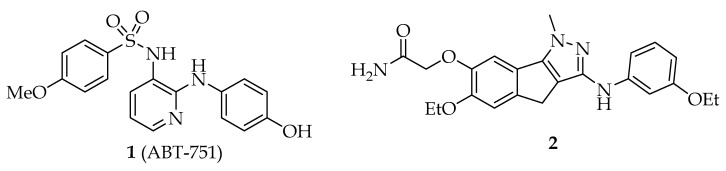
Tubulin inhibitors **1** and **2**.

*N*-(2-((4-Hydroxyphenyl)amino)pyridin-3-yl)-4-methoxybenzenesulfonamide (ABT-751, **1**, Figure 1) is an orally available sulfonamide tubulin inhibitor under clinical investigations for the treatment of cancers [24,25]. On its X-ray crystal structures with tubulin, ABT-751 interacted with all the three pockets of tubulin at the colchicine binding site [26]. Based on the binding mode of **1** with tubulin and the pyrazole pharmacophore, our group designed and synthesized a series of indenopyrazoles as potential tubulin polymerization inhibitors targeting the colchicine binding site [27]. The indenopyrazole analogue **2** (Figure 1) was found to compete with colchicine in binding to the tubulin colchicine site and inhibit the polymerization of tubulin. In vitro, **2** displayed nanomolar potency against a variety of tumor cell lines, arrested tumor cells in G2/M phase through the regulation of cell cycle-related proteins, and induced tumor cell apoptosis through the activation of caspase pathways. Furthermore, **2** was effective for multidrug resistance tumor cells and inhibited phosphatase and tensin homolog (PTEN) phosphorylation and PTEN/Akt/NF-кB signaling [28]. In vivo, **2** demonstrated its potency in non-small cell lung cancer (NSCLC) and vincristine-resistance human oral epidermoid carcinoma cell (KB/V) xenograft models without obvious side effects [27,28]. In this study, we replaced the indenopyrazole core with the 1*H*-benzofuro[3,2-*c*]pyrazole framework and designed a series of the 1*H*-benzofuro[3,2-*c*]pyrazole derivatives **4a**–**e**. In the preparation of **4a**–**e**, the partial cleavage of the furan ring was observed and a series of 5-methoxy-2-(3-(phenylamino)-1H-pyrazol-5-yl)phenol derivatives **5a**–**e** were isolated. We reported here the synthesis and preliminary results of their tumor cell growth inhibitory activity as well as the identification of pyrazole **5b** as a novel tubulin polymerization inhibitor.

## 2. Results and Discussion

### 2.1. Chemistry

**Scheme 1:**
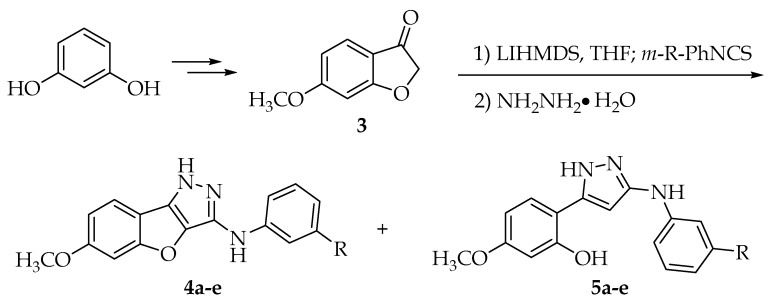
Synthesis of benzofuropyrazole and pyrazole derivatives.

The synthetic route towards the benzofuropyrazole derivatives was shown in Scheme 1. The 6-methoxybenzofuran-3-(2*H*)-one **3** was prepared according to the reported method in three steps [29,30,31] (please refer to the Supplementary Materials). The Hoesch reaction of resorcinol with chloroacetonitrile in the presence of anhydrous ZnCl_2_ and HCl gas generated an imine intermediate, which upon hydrolysis, provided 2-chloro-1-(2,4-dihydroxyphenyl)ethanone in 94% yield. Treatment of the chloromethyl ketone with a mild base, CH_3_COONa, gave the cyclized 6-hydroxybenzofuran-3-(2*H*)-one (54%). Methylation of 6-hydroxybenzofuran-3-(2*H*)-one with Me_2_SO_4_ produced the 6-methoxybenzofuran-3-(2*H*)-one **3** in 86% yield. After the deprotonation of the α-proton of carbonyls in **3** with lithium hexamethyldisilazide (LiHMDS), the resulting enolates were reacted with 3-substitued phenyl isothiocyanates to give the thioamide intermediates, which underwent condensation with hydrazine monohydrate in dioxane/EtOH (1:1) to form the benzofuropyrazole derivatives **4a**–**e** in 11% to 30% yield. In this process, the partial cleavage of the furan ring occurred, a series of 5-methoxy-2-(3-(phenylamino)-1*H*-pyrazol-5-yl)phenol derivatives **5a**–**e** were also isolated in 13% to 31% yield. The structures of benzofuropyrazoles **4a**–**e** and pyrazoles **5a**–**e** were determined by ^1^H nuclear magnetic resonance (NMR), ^13^C-NMR and electrospray ionization mass spectrometry (ESI-MS). In the ^1^H NMR spectrum of benzofuropyrazole **4c**, the pyrazole 1-NH appeared at 11.91 ppm, the aniline NH at 8.05 as a singlet, the amide NH at 8.28 (q, *J* = 4.6 Hz) in corresponding with the N-methyl at 2.77 (d, *J* = 4.6 Hz). The seven protons at the two phenyl rings appeared at 6.42 to 7.82 ppm. In comparison with **4c**, in the ^1^H NMR spectrum of pyrazole **5c**, there were two additional peaks at 10.23 ppm for the phenol OH, 6.24 ppm for the pyrazole 4-H, supporting its estimated structure.

### 2.2. Tumor Cell Growth Inhibitory Activity

All the synthesized compounds were evaluated for their tumor cell growth inhibitory activity against human breast cancer MCF-7 cell, human erythroleukemia K562 cell and human lung cancer A549 cell by the conventional MTT (3-(4,5-dimethyl-2-thiazolyl)-2,5-diphenyl-2*H*-tetrazolium bromide) assay. ABT-751 was used as positive control.

As shown in Table 1, the breast tumor MCF-7 cell was not sensitive to the benzofuropyrazole derivatives **4a**–**e**. For the K562 and A549 cells, only **4a** exhibited high potency and was more potent than ABT-71 with the GI_50_ of 0.26 and 0.19 μM, respectively, while other benzofuropyrazoles showed moderate or weak activity. It seems the substitution of the ethoxy at the aniline ring with the electron-withdrawing ester, amide, and cyano groups was not tolerated among the benzofuropyrazole series. In contrast, all the three tumor cell lines were sensitive to the pyrazole analogues **5a**–**e**. The methyl ester **5b** was the most active that inhibited the K562, MCF-7, and A549 cell growth with GI_50_ values of 0.021, 1.7 and 0.69 μM, respectively. Both compounds **5a** and **5b** were highly active against the K562 and A549 cells, and were 5- to 35-fold more potent than ABT-751. The cyano derivative **5e** was also highly potent against the three tumor cell lines, although it showed slightly less potency than ABT-751. Unlike the benzofuropyrazole derivatives, the substitution of the ethoxy at the aniline ring with an electron-withdrawing ester, amide, and cyano group in the pyrazole series was well tolerated, and even preferred, indicating that the benzofuropyrazole derivatives **4a**–**e** and the pyrazoles **5a**–**e** might involve different mechanisms of action. 

### 2.3. In Vitro Tubulin Polymerization Inhibitory Activity 

**Figure 2:**
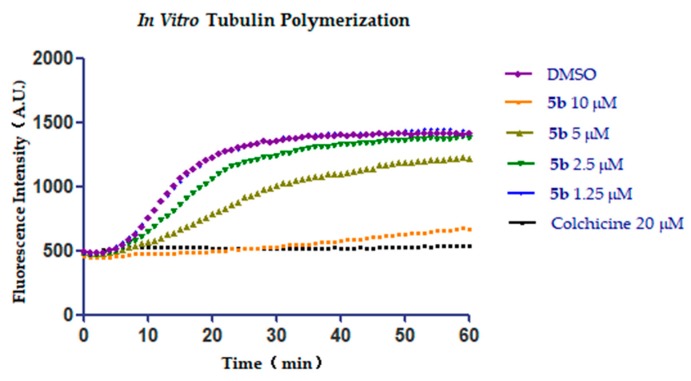
Effect of **5b** on tubulin polymerization in vitro. Purified tubulin protein at 2 mg/mL in a reaction buffer was incubated at 37 °C in the presence of 1% dimethyl sulfoxide (DMSO), test compound **5b** at 1.25, 2.5, 5, or 10 μM or colchicine at 20 μM. The fluorescence intensity was measured every 60 s for 60 min and is presented as increases in the polymerized microtubule.

The pyrazole derivative **5b** showed the best tumor cell growth inhibitory activity among all the tested compounds. To investigate whether **5b** was a tubulin inhibitor, the tubulin polymerization inhibition assay was carried out. At 37 °C, tubulin will polymerize into microtubules, which is followed by the observed fluorescence enhancement due to the incorporation of a fluorescent reporter into microtubules as polymerization occurs [32]. As shown in Figure 2, **5b** inhibited the tubulin polymerization in a concentration-dependent way with a calculated IC_50_ of 7.30 μM. Therefore, **5b** may be a good lead for further structural modification to find more potent tubulin inhibitors based on the privileged pyrazole structure.

## 3. Materials and Methods

### 3.1. General Chemical Experimental Procedures

Melting points were determined on an X-6 micromelting point apparatus (Beijing Tech. Co., Ltd.). ^1^H- and ^13^C-NMR spectra were recorded on Bruker-400 NMR or Bruker-600 NMR spectrometers. All spectra were recorded at room temperature for DMSO or CDCl_3_ solutions. ESI-MS was performed on an API 4000 instrument. Thin-layer chromatography (TLC) was performed on silica gel GF254 plates. Silica gel GF254 and silica gel (200−300 mesh) from Qingdao Haiyang Chemical Company were used for TLC and column chromatography, respectively. All reagents were commercially available and were used as purchased without further purification. All reactions involving oxygen- or moisture sensitive compounds were carried out under a dry N_2_ atmosphere. Unless otherwise noted, reagents were added by syringe. Tetrahydrofuran (THF) was distilled from sodium/benzophenone immediately prior to use.

*3-((6-Methoxy-1H-benzofuro[3,2-c]pyrazol-3-yl)amino)ethoxybenzene* (**4a**) *and 3-((5-(2-hydroxy-4-methoxyphenyl)-1H-pyrazol-3-yl)amino)ethoxybenzene* (**5a**) A solution of 6-methoxybenzofuran-3-(2H)-one **3** (150 mg, 0.9 mmol) in anhydrous THF (5 mL) was cooled to −78 °C under nitrogen atmosphere. LiHMDS (1.09 mL, 1.1 mmol, 1.0 M THF solution) was added dropwise. The mixture was stirred at −78 °C for 2 h and then warmed to −45 °C in 45 min. After a solution of the 1-ethoxy-3-isothiocyanatobenzene (164 mg, 1.0 mmol) in anhydrous THF (3 mL) was added, the resulting mixture was stirred at room temperature overnight. Water (30 mL) was added, and the mixture was extracted with EtOAc (3 × 30 mL). The organic layer was washed with brine, dried over anhydrous Na_2_SO_4_. After filtration and evaporation, flash column chromatography on silica gel (hexane/EtOAc = 15:1) gave the resulting thioamide intermediate, which was dissolved in 1,4-dioxane (3 mL) and ethanol (3 mL). Hydrazine hydrate (0.46 mL, 7.3 mmol) was added dropwise. The mixture was heated to 50 °C and stirred for 24 h. Water (40 mL) was added, and the mixture was extracted with EtOAc (3 × 40 mL). The organic layer was washed with brine, and dried over anhydrous Na_2_SO_4_. After filtration and evaporation, the residue was purified by column chromatography on silica gel (hexane/EtOAc = 4:1) to give **4a** (32 mg, 11%) and **5a** (38 mg, 13%).

**4a**: Brown solid, m.p.: 62–64 °C. ^1^H-NMR (CDCl_3_) δ 7.32 (d, *J* = 8.6 Hz, 1H, Ar-H), 7.08 (t, *J* = 8.1 Hz, 1H, Ar-H), 6.56 (d, *J* = 2.4 Hz, 1H, Ar-H), 6.46 (dd, *J* = 2.4, 8.6 Hz, 1H, Ar-H), 6.36 (d, *J* = 8.2 Hz, 1H, Ar-H), 6.25 (d, *J* = 8.1 Hz, 1H, Ar-H), 6.21 (s, 1H, Ar-H), 5.09 (s, 1H, 3-NH), 3.96 (q, *J* = 6.9 Hz, 2H, OCH_2_), 3.81 (s, 3H, 6’-OCH_3_), 1.37 (t, *J* = 6.9 Hz, 3H, OCH_2_CH_3_). ^13^C-NMR (CDCl_3_) δ 161.6, 160.3, 156.6, 146.0, 137.8, 130.2, 129.6, 127.9, 125.8, 111.5, 109.5, 107.0, 105.1, 103.7, 101.0, 63.3, 55.3, 14.9. MS (ESI) calcd. for C_18_H_19_N_3_O_3_ [M + NH_4_]^+^: 341.1, found: 341.4.

**5a**: Brown solid, m.p.: 82–84 °C. ^1^H-NMR (CDCl_3_) δ 7.39 (d, *J* = 8.6 Hz, 1H, Ar-H), 7.17 (t, *J* = 8.0 Hz, 1H, Ar-H), 6.57–6.49 (m, 5H, Ar-H), 6.22 (s, 1H, 4’-H), 5.79 (s, 1H, 3-NH), 4.01 (q, *J* = 6.9 Hz, 2H, 1-OCH_2_), 3.79 (s, 3H, 4’’-OCH_3_), 1.41 (t, *J* = 6.9 Hz, 3H, 1-CH_3_). ^13^C-NMR (CDCl_3_) δ 160.7, 160.2, 157.0, 143.9, 130.4, 127.6, 109.8, 108.7, 107.1, 106.5, 102.8, 101.7, 90.4, 63.3, 55.3, 15.4. MS (ESI) calcd. for C_18_H_19_N_3_O_3_ [M + H]^+^: 326.1, found: 326.4.

*Methyl 3-((6-methoxy-1H-benzofuro[3,2-c]pyrazol-3-yl)amino)benzoate* (**4b**) *and methyl 3-((5-(2-hydroxy-4-methoxyphenyl)-1H-pyrazol-3-yl)amino)benzoate* (**5b**) According to the procedures described for the synthesis of **4a** and **5a**, compounds **4b** and **5b** were prepared from **3** (100 mg, 0.6 mmol), LiHMDS (0.7 mL, 0.7 mmol), methyl 3-isothiocyanatobenzoate (135 mg, 0.7 mmol) and hydrazine hydrate (0.3 mL, 4.8 mmol). The crude residue was purified by column chromatography on silica gel (hexane/EtOAc = 3:1) to give **4b** (36 mg, 18%) and **5b** (33 mg, 16%).

**4b**: Brown solid, m.p.: 103–105 °C. ^1^H-NMR (DMSO-*d*_6_) δ 11.96 (s, 1H, 1’-NH), 8.19 (s, 1H, 3-NH), 8.13 (s, 1H, Ar-H), 7.61 (s, 1H, Ar-H), 7.43 (d, *J* = 8.3 Hz, 1H, Ar-H), 7.33 (d, *J* = 4.3 Hz, 2H, Ar-H), 6.50 (dd, *J* = 1.9, 8.5 Hz, Ar-H), 6.42 (d, *J* = 2.0 Hz, 1H, Ar-H), 3.84 (s, 3H, COOCH_3_), 3.74 (s, 3H, OCH_3_). ^13^C-NMR (DMSO-*d*_6_) δ 167.2, 161.1, 156.9, 145.9, 144.6, 134.1, 130.6, 129.6, 128.3, 119.8, 119.2, 115.6, 110.4, 109.1, 106.2, 103.6, 55.5, 52.5. MS (ESI) calcd. for C_18_H_15_N_3_O_4_ [M + NH_4_]^+^: 355.1, found: 355.5.

**5b**: Yellow solid, m.p.: 185–186 °C. ^1^H-NMR (DMSO-*d*_6_) δ 12.01 (s, 1H, 1’-NH), 10.24 (s, 1H, 2’’-OH), 8.67 (s, 1H, 3-NH), 8.13 (s, 1H, Ar-H), 7.57 (s, 1H, Ar-H), 7.53 (d, *J* = 6.8 Hz, 1H, Ar-H), 7.31 (s, 2H, Ar-H), 6.51 (s, 1H, Ar-H), 6.50 (dd, *J* = 1.9, 6.9 Hz, 1H, Ar-H), 6.23 (s, 1H, 4’-H), 3.84 (s, 3H, COOCH_3_), 3.74 (s, 3H, OCH_3_). ^13^C-NMR (DMSO-*d*_6_) δ 167.2, 160.3, 155.8, 151.6, 144.8, 139.8, 130.7, 129.4, 128.4, 119.7, 118.8, 115.5, 110.0, 105.8, 102.1, 93.0, 55.5, 52.2. MS (ESI) calcd. for C_18_H_17_N_3_O_4_ [M + H]^+^: 340.1, found: 340.4. 

*3-((6-Methoxy-1H-benzofuro[3,2-c]pyrazol-3-yl)amino)-N-methylbenzamide* (**4c**) *and 3-((5-(2-hydroxy-4-methoxyphenyl)-1H-pyrazol-3-yl)amino)-N-methylbenzamide* (**5c**) According to the procedures described for the synthesis of **4a** and **5a**, compounds **4c** and **5c** were prepared from **3** (100 mg, 0.6 mmol), LiHMDS (0.7 mL, 0.7 mmol), 3-isothiocyanato-N-methylbenzamide (134 mg, 0.7 mmol) and hydrazine hydrate (0.3 mL, 4.8 mmol). The crude residue was purified by column chromatography on silica gel (hexane/EtOAc = 1:1) to give **4c** (30 mg, 15%) and **5c** (33 mg, 16%).

**4c**: Brown solid, m.p.: 180–182 °C. ^1^H-NMR (DMSO-*d*_6_) δ 11.91 (s, 1H, 1’-NH), 8.28 (q, *J* = 4.6 Hz, 1H, CONH), 8.05 (s, 1H, 3-NH), 7.82 (s, 1H, Ar-H), 7.55 (s, 1H, Ar-H), 7.43 (d, *J* = 5.7 Hz, 1H, Ar-H), 7.27 (t, *J* = 7.8 Hz, 1H, Ar-H), 7.15 (d, *J* = 7.6 Hz, 1H, Ar-H), 6.49 (dd, *J* = 2.6, 8.6 Hz, 1H, Ar-H), 6.42 (d, *J* = 2.6 Hz, 1H, Ar-H), 3.74 (s, 3H, OCH_3_), 2.77 (d, *J* = 4.6 Hz, 3H, N-CH_3_). ^13^C-NMR (DMSO-*d*_6_) δ 167.8, 161.1, 156.9, 146.1, 144.5, 135.9, 134.2, 129.1, 128.3, 117.4, 116.7, 114.5, 110.5, 109.1, 106.1, 103.6, 55.5, 26.7. MS (ESI) calcd. for C_18_H_16_N_4_O_3_ [M + NH_4_]^+^: 354.1, found: 354.4.

**5c**: Brown solid, m.p.: 184–186 °C. ^1^H-NMR (DMSO-*d*_6_) δ 11.94 (s, 1H, 1’-NH), 10.23 (s, 1H, OH), 8.52 (q, *J* = 3.6 Hz, 1H, CONH), 8.25 (s, 1H, 3-NH), 7.81 (s, 1H, Ar-H), 7.52 (s, 2H, Ar-H), 7.25 (s, 1H, Ar-H), 7.11 (s, 1H, Ar-H), 6.51 (s, 1H, Ar-H), 6.49 (dd, *J* = 1.6, 6.9 Hz, 1H, Ar-H), 6.24 (s, 1H, 4’-H), 3.74 (s, 3H, OCH_3_), 2.76 (d, *J* = 3.6 Hz, 3H, N-CH_3_). ^13^C-NMR (DMSO-*d*_6_) δ 170.0, 160.3, 155.8, 151.8, 144.6, 139.7, 136.1, 129.0, 128.4, 117.6, 116.4, 114.3, 110.2, 105.8, 102.1, 93.0, 55.5, 26.7. MS (ESI) calcd. for C_18_H_18_N_4_O_3_ [M + H]^+^: 339.1, found: 339.1. 

*3-((6-Methoxy-1H-benzofuro[3,2-c]pyrazol-3-yl)amino)benzamide* (**4d**) *and 3-((5-(2-hydroxy-4-methoxyphenyl)-1H-pyrazol-3-yl)amino)benzamide* (**5d**) According to the procedures described for the synthesis of **4a** and **5b**, compounds **4d** and **5d** were prepared from **3** (100 mg, 0.6 mmol), LiHMDS (0.7 mL, 0.7 mmol), 3-isothiocyanatobenzamide (124 mg, 0.7 mmol) and hydrazine hydrate (0.3 mL, 4.8 mmol). The crude residue was purified by column chromatography on silica gel (hexane/EtOAc = 1:3) to give **4d** (59 mg, 30%) and **5d** (61 mg, 31%).

**4d**: Brown solid, m.p.: 172–174 °C. ^1^H-NMR (DMSO-*d*_6_) δ 12.02 (s, 1H, 1’-NH), 8.12 (s, 1H, 3-NH), 7.82 (s, 2H, Ar-H), 7.52 (s, 1H, Ar-H), 7.43 (d, *J* = 2.9 Hz, 1H, Ar-H), 7.26–7.21 (m, 3H, 1-CONH_2_, Ar-H), 6.51 (d, *J* = 5.7 Hz, 1H, Ar-H), 6.44 (s, 1H, Ar-H), 3.74 (s, 3H, OCH_3_). ^13^C-NMR (DMSO-*d*_6_) δ 169.0, 161.0, 156.9, 146.1, 144.5, 135.6, 134.2, 129.0, 128.3, 117.7, 117.2, 114.8, 110.5, 109.2, 106.1, 103.6, 55.5. MS (ESI) calcd. for C_17_H_14_N_4_O_3_ [M + NH_4_]^+^: 340.1, found: 340.1.

**5d**: Brown solid, m.p.: 128–130 °C. ^1^H-NMR (DMSO-*d*_6_) δ 11.84 (s, 1H, 1’-NH), 8.56 (s, 1H, 3-NH), 7.83 (s, 1H, Ar-H), 7.75 (s, 1H, Ar-H), 7.54 (d, *J* = 6.8 Hz, 1H, Ar-H), 7.43 (s, 1H, Ar-H), 7.26-7.21 (m, 3H, 1-CONH_2_, Ar-H), 6.51 (s, 1H, Ar-H), 6.49 (d, *J* = 6.8 Hz, 1H, Ar-H), 6.29 (s, 1H, 4’-H), 3.74 (s, 3H, OCH_3_). ^13^C-NMR (DMSO-*d*_6_) δ 169.1, 160.3, 144.5, 135.8, 129.1, 128.3, 117.8, 114.6, 110.3, 105.8, 102.1, 55.5. MS (ESI) calcd. for C_17_H_16_N_4_O_3_ [M + H]^+^: 325.1, found: 325.1. 

*3-((6-Methoxy-1H-benzofuro[3,2-c]pyrazol-3-yl)amino)benzonitrile* (**4e**) *and 3-((5-(2-hydroxy-4-methoxyphenyl)-1H-pyrazol-3-yl)amino)benzonitrile* (**5e**) According to the procedures described for the synthesis of **4a** and **5b**, compounds **4e** and **5e** were prepared from **3** (100 mg, 0.6 mmol), LiHMDS (0.7 mL, 0.7 mmol), 3-isothiocyanatobenzonitrile (112 mg, 0.7 mmol) and hydrazine hydrate (0.3 mL, 4.8 mmol). The crude residue was purified by column chromatography on silica gel (hexane/EtOAc = 1:4) to give **4e** (31 mg, 17%) and **5e** (33 mg, 18%). 

**4e**: White solid, m.p.: 200–202 °C. ^1^H-NMR (DMSO-*d*_6_) δ 12.01 (s, 1H, 1’-NH), 8.39 (s, 1H, 3-NH), 7.91( s, 1H, Ar-H), 7.53(d, *J* = 6.9 Hz, 1H, Ar-H), 7.43-7.38 (m, 2H, Ar-H), 7.15 (d, *J* = 7.4 Hz, 1H, Ar-H), 6.51 (dd, *J* = 2.5, 8.6 Hz, 1H, Ar-H), 6.44 (d, *J* = 2.5 Hz, 1H, Ar-H), 3.75 (s, 3H, OCH_3_). ^13^C-NMR (DMSO-*d*_6_) δ 161.1, 156.9, 145.2, 145.0, 134.0, 130.5, 128.5, 121.7, 120.0, 119.9, 117.2, 112.0, 110.3, 109.7, 106.2, 103.6, 55.5. MS (ESI) calcd. for C_17_H_12_N_4_O_2_ [M + NH_4_]^+^: 322.1, found: 322.1.

**5e**: White solid, m.p.: 166–168 °C. ^1^H-NMR (DMSO-*d*_6_) δ 12.05 (s, 1H, 1’-NH), 10.28 (s, 1H, OH), 8.93 (s, 1H, 3-NH), 7.99 (s, 1H, Ar-H), 7.55 (s, 1H, Ar-H), 7.53 (s, 1H, Ar-H), 7.38 (s, 1H, Ar-H), 7.12 (d, *J* = 4.4 Hz, 1H, Ar-H), 6.53 (s, 1H, Ar-H), 6.51 (dd, *J* = 1.3, 5.7 Hz, 1H, Ar-H), 6.25 (s, 1H, 4’-H), 3.78 (s, 3H, OCH_3_). ^13^C-NMR (DMSO-*d*_6_) δ 160.4, 155.8, 151.2, 145.0, 140.0, 130.4, 128.5, 121.3, 120.0, 117.3, 112.0, 109.8, 105.8, 102.1, 92.8, 55.5. MS (ESI) calcd. for C_17_H_14_N_4_O_2_ [M + H]^+^: 307.1, found: 307.3. 

### 3.2. MTT Assay

The human tumor cell lines, were grown in Roswell Park Memorial Institute (RPMI) 1640 medium and supplemented with 10% foetal bovine serum in the 37 °C in an atmosphere containing 5% CO_2_. All the synthesized compounds were assayed by conventional 3-(4,5-dimethyl-2-thiazolyl)-2,5-diphenyl-2*H*-tetrazolium bromide (MTT) method. In brief, the exponentially growing cells were seeded into 96-well cell plates at a density of 4−4.5 × 10^3^ cells per well and allowed to adhere overnight. Cells were incubated with various concentrations of the test compounds for 72 h. Then 20 μL of MTT (2.5 mg/mL) was added, the cells were incubated at 37 °C for another 4 h. The reduced MTT crystals were dissolved in DMSO, and the absorbance was measured at 570 nm by a microplate spectrophotometer. The growth in inhibitory effects of each compound were expressed as GI_50_ values, which represent the molar drug concentrations required to cause 50% tumor cell growth inhibition.

### 3.3. In Vitro Tubulin Polymerization Inhibition Assay

The fluorescence-based in vitro tubulin polymerization assay was performed using the Tubulin Polymerization Assay Kit (BK011P, Cytoskeleton, USA) according to the manual. The tubulin reaction mix contained 2 mg/mL porcine brain tubulin (>99% pure), 2 mM MgCl_2_, 0.5 mM ethylene glycol-bis(2-aminoethylether)-*N,N,N′,N′*-tetraacetic acid (EGTA), 1 mM guanosine triphosphate (GTP), and 15% glycerol. First, a 96-well plate was incubated with 5 μL of inhibitors in different concentrations at 37 °C for 1 min. Then 50 μL of the tubulin reaction mix was added. Immediately, the increase in fluorescence was monitored by excitation at 355 nm and emission at 460 nm in a multimode reader.

## 4. Conclusions

In the synthesis of 1*H*-benzofuro[3,2-*c*]pyrazole derivatives **4a**–**e**, the furan ring-opening was observed, and a series of pyrazole derivatives **5a**–**e** were identified. In the tumor cell growth inhibitory assay, only **4a** was highly active towards the K562 and A549 cells, while other benzofuropyrazole derivatives were not active or showed weak activity. In general, the pyrazoles **5a-e** were more potent than the corresponding benzofuropyrazole derivatives. Compound **5a** exhibited a similar tendency to that of **4a** with high potency against K562 and A549 cells but weak effects on MCF-7 cells. Both pyrazoles **5b** and **5e** exhibited high inhibitory activities against K562, MCF-7 and A549 cells. The most active compound **5b** was 5- to 35-fold more potent than ABT-751 in the inhibition of A549 and K562 cells. In addition, **5b** inhibited tubulin polymerization inhibition with an IC_50_ of 7.30 μM. These results indicated that **5b** was a novel tubulin polymerization inhibitor and it may be a good lead for the discovery of novel pyrazoles as potent anticancer agents.

## Figures and Tables

**Table 1:** Tumor cell growth inhibitory activity of **4a**–**e** and **5a**–**e**.

		GI_50_ (μM)		
Compound	R	K562	MCF-7	A549
**4a**	OCH_2_CH_3_	0.26 ± 0.04	>20	0.19 ± 0.08
**4b**	COOCH_3_	5.46 ± 1.04	>20	>20
**4c**	CONHCH_3_	5.11 ± 0.31	>20	15.11 ± 2.18
**4d**	CONH_2_	9.01 ± 1.81	>20	10.08 ± 2.21
**4e**	CN	13.53 ± 0.41	>20	17.01 ± 2.76
**5a**	OCH_2_CH_3_	0.046 ± 0.007	16.72 ± 2.6	0.92 ± 0.17
**5b**	COOCH_3_	0.021 ± 0.004	1.7 ± 0.43	0.69 ± 0.18
**5c**	CONHCH_3_	7.33 ± 1.004	7.78 ± 0.87	9.46 ± 2.03
**5d**	CONH_2_	14.77 ± 2.62	5.8 ± 0.202	10.9 ± 0.99
**5e**	CN	1.45 ± 0.047	2.27 ± 0.34	3.24 ± 0.99
ABT-751		0.74 ± 0.078	0.88 ± 0.24	4.58 ± 0.04

## Supplementary Materials

Supplementary materials are available online.

## Abbreviations

THF	Tetrahydrofuran
MCF-7	Breast cancer cell
K562	Human erythroleukemia cell
A549	Lung cancer cell
NSCLC	Non-small cell lung cancer
KB/V	Vincristine-resistance human oral epidermoid carcinoma cell
LiHMDS	Lithium bis(trimethylsilyl)amide
PTEN	Phosphatase and tensin homolog
DMSO	Dimethyl sulfoxide
RPMI	Roswell park memorial institute
EGTA	Ethylene glycol-bis(2-aminoethylether)-*N,N,N‘,N’*-tetraacetic acid
GTP	Guanosine triphosphate
