# Supplementary material for: Synthesis of Novel Pyrazole Derivatives and Their Tumor Cell Growth Inhibitory Activity
Source: Molecules. 2019 Jan 13;24(2):279. doi: 10.3390/molecules24020279 (PMC6359563; doi:10.3390/molecules24020279)
Supplement: Supplementary file 1 [file molecules-24-00279-s001.pdf]

Synthesis of Novel Pyrazole Derivatives and Their Tumor Cell Growth Inhibitory Activity

Ying-Jie Cui, Long-Qian Tang, Cheng-Mei Zhang, Zhao-Peng Liu*

Institute of Medicinal Chemistry, Key Laboratory of Chemical Biology (Ministry of Education), School of Pharmaceutical Sciences, Shandong University, Jinan 250012, PR China

* Correspondence: liuzhaop@sdu.edu.cn; Tel. +86-531-88382006; Fax: +86-531-88382548

Synthesis of intermediates ……………………………………………………………………2

^{1}H and ^{13}C NMR spectra for the synthesized compounds……………………….…………3-12

Synthesis of intermediates

2-Chloro-1-(2,4-dihydroxyphenyl)ethan-1-one

To a mixture of resorcinol (500 mg, 4.5 mmol) and chloroacetonitrile (0.35 mL, 5.5 mmol) in ether (10 mL) was added anhydrous $ZnCl_2$ (300 mg, 2.3 mmol). The solution was cooled to 0 °C and HCl gas was bubbled through the reaction mixture for 2 h. The solution was left in 4 °C overnight and HCl gas was bubbled into the reaction mixture again for 2 h. Then the solution was left in the 4 °C for 2 days. The precipitated imine was filtered off and washed three times with ether. The imine was dissolved in 15 mL of water and reflux for 1 h. The solution was extracted with EtOAc (3 × 45 mL), washed with brine (30 mL), and dried over Na_2SO_4. After filtration and evaporation, the residue was purified by column chromatography on silica gel (hexane/EtOAc = 1:4) to afford pure 2-chloro-1-(2,4-dihydroxyphenyl)ethan-1-one as a white solid. Yield 790 mg (94%), m.p.: 106-107 °C. ^{1}H-NMR (DMSO-d_6) δ 11.67 (s, 1H), 10.69 (s, 1H), 7.74 (d, *J* = 7.1 Hz, 1H), 6.41 (dd, *J* = 1.8, 7.0 Hz, 1H), 6.34 (d, *J* = 1.8 Hz, 1H), 5.01 (s, 2H). ^{13}C-NMR (DMSO-d_6) δ 193.7, 165.9, 164.0, 133.3, 112.1, 109.1, 103.2, 48.3. MS (ESI) $[M + H]^+$: 164.99.

6-Hydroxybenzofuran-3(2H)-one

To a solution of 2-chloro-1-(2,4-dihydroxyphenyl)ethan-1-one (160 mg, 0.86 mmol) in EtOH (5 mL), sodium acetate (85 mg, 1.03 mmol) was added. The mixture was heated to 60 °C and stirred vigorously for 5 h. The solution was evaporated and water (25 mL) was added. The resulting precipitate was filtered and dried to give 70 mg (54%) of 6-hydroxybenzofuran-3(2H)-one as a white solid, m.p.: >220 °C. 1H-NMR (DMSO-d_6) δ 10.93 (s, 1H), 7.47 (d, *J* = 6.8 Hz, 1H), 6.59 (d, *J* = 1.6, 6.8 Hz, 1H), 6.51 (d, *J* = 1.5 Hz, 1H), 6.34 (d, *J* = 1.8 Hz, 1H), 4.71 (s, 2H). ^{13}C-NMR (DMSO-d_6) δ 197.3, 176.1, 167.2, 125.5, 113.4, 112.2, 98.8, 75.7. MS (ESI) $[M - H]^-$: 185.05.

6-Methoxybenzofuran-3(2H)-one

To a mixture of 6-hydroxybenzofuran-3(2*H*)-one (60 mg, 0.4 mmol) and K_2CO_3 (56 mg, 0.4 mmol) in DMF (3 mL), dimethyl sulfate (43 μL, 0.4 mmol) was added. The mixture was stirred vigorously for 50 min at room temperature. The water (30 mL) was added, and the mixture was extracted with EtOAc (3 × 20 mL). The organic layer was washed with brine (40 mL), dried over anhydrous Na_2SO_4, and concentrated. The residue was purified by column chromatography on silica gel (hexane/EtOAc = 1:6) to give 56 mg (86%) of 6-methoxybenzofuran-3(2H)-one as a light yellow solid, m.p.: 112–114 °C. ^{1}H-NMR (DMSO-d_6) δ 7.54 (d, *J* = 6.9 Hz, 1H), 6.82 (d, *J* = 1.6 Hz, 1H), 6.72 (dd, *J* = 1.6, 6.9 Hz, 1H), 4.77 (s, 2H), 3.87 (s, 3H). ^{13}C-NMR (DMSO-d_6) δ 197.6, 176.3, 168.2, 125.1, 114.5, 112.0, 97.2, 76.0, 56.6. MS (ESI) $[M - H]^-$: 149.06.

^{1}H and ^{13}C NMR spectra for the synthesized compounds

Compound 4a

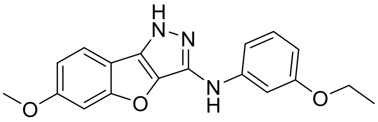

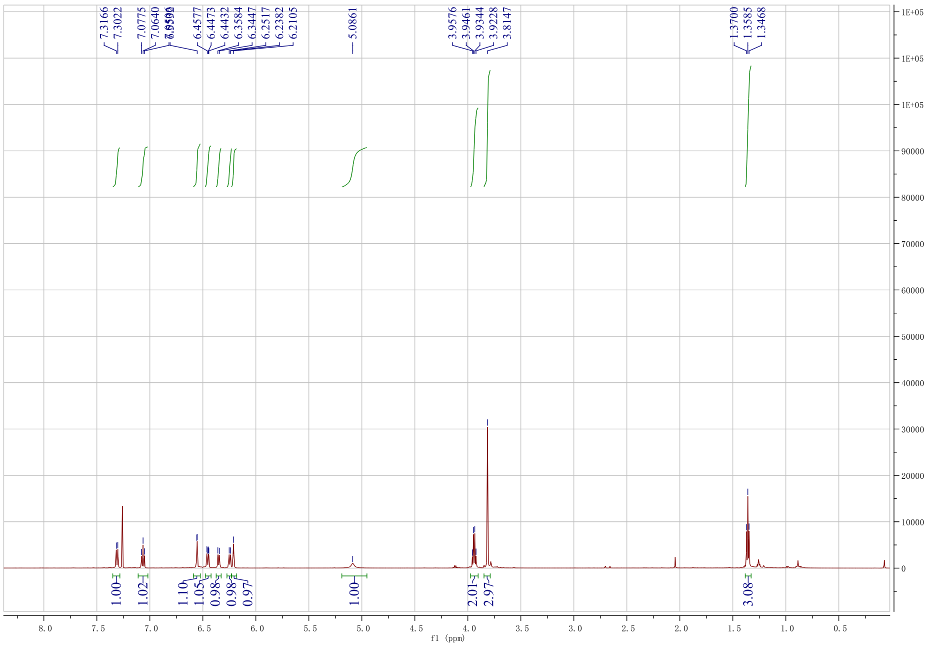

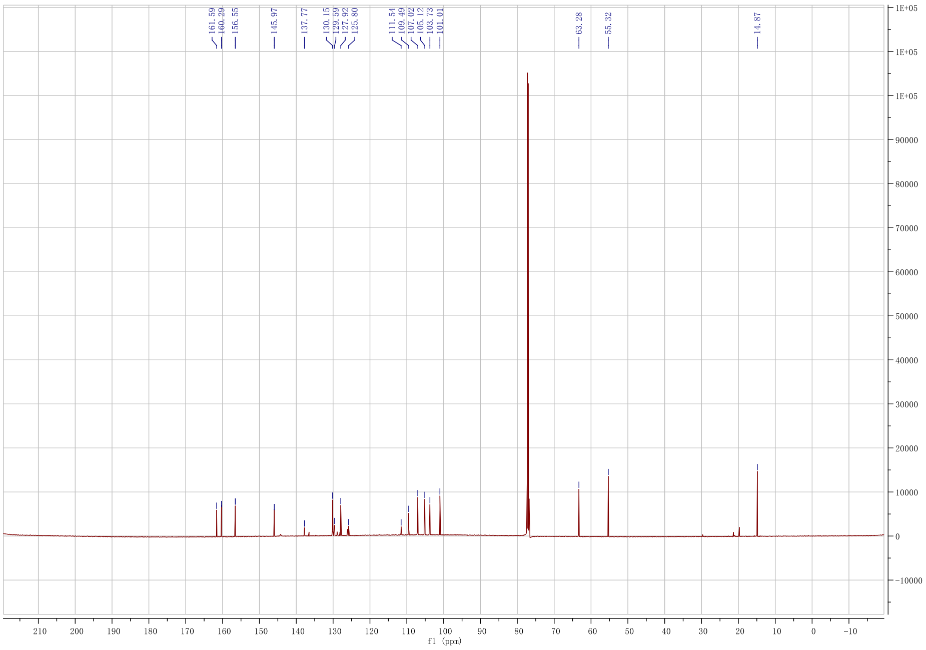

Compound 4b

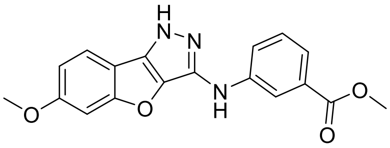

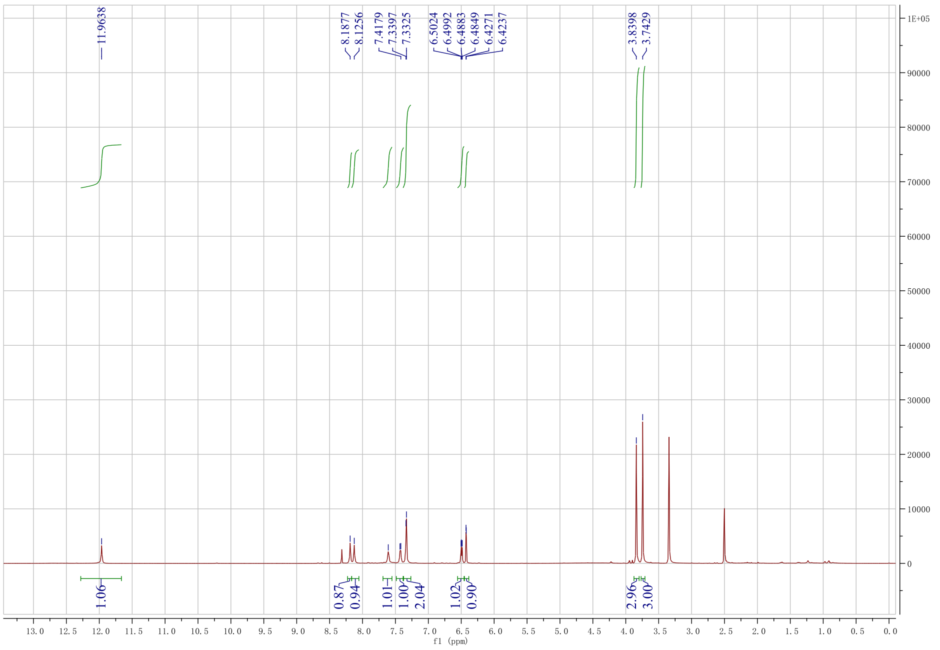
11.9638
8.1877
8.1256
7.4179
7.3397
7.3325
6.5024
6.4992
6.4883
6.4849
6.4271
6.4237
3.8398
3.7429
1.06
0.87
0.94
1.01
1.00
2.04
1.02
0.90
2.96
3.00
f1 (ppm)

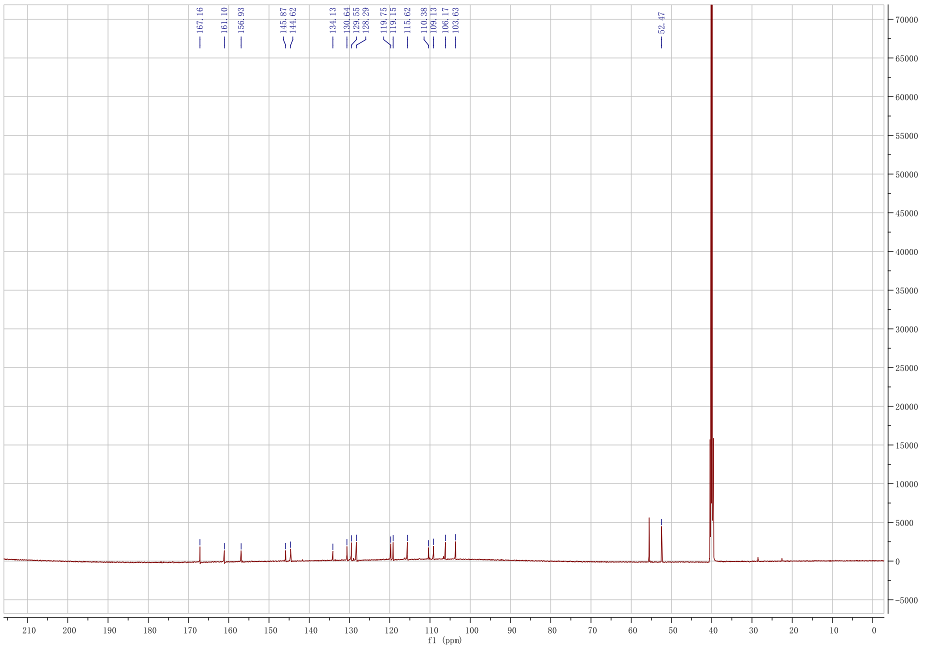
167.16
161.10
156.93
145.87
144.62
134.13
130.64
129.55
128.29
119.75
119.15
115.62
110.38
109.13
106.17
103.63
52.47
f1 (ppm)

Compound 4c

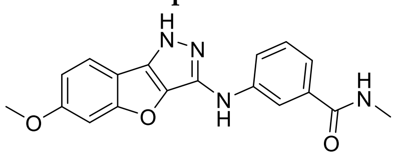

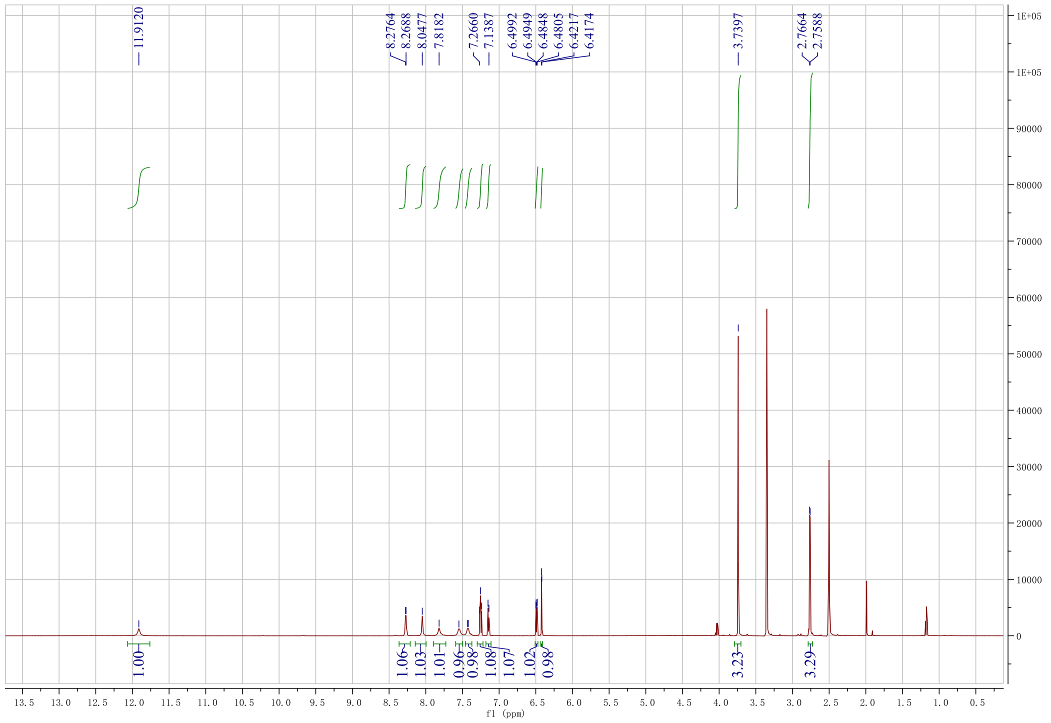
11.9120
8.2764
8.2688
8.0477
7.8182
7.2660
7.1387
6.4992
6.4949
6.4848
6.4805
6.4217
6.4174
3.7397
2.7664
2.7588
1.00
1.06
1.03
1.01
0.96
0.98
1.08
1.07
1.02
0.98
3.23
3.29
f1 (ppm)

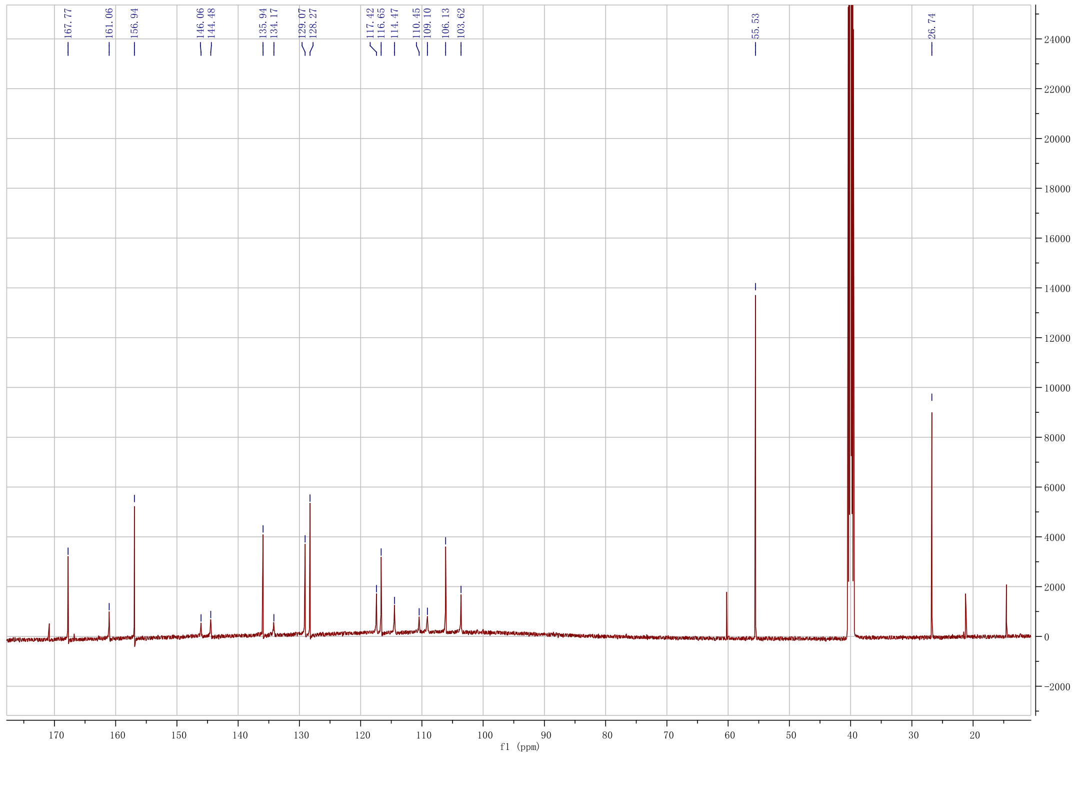
167.77
161.06
156.94
146.06
144.48
135.94
134.17
129.07
128.27
117.42
116.65
114.47
110.45
109.10
106.13
103.62
55.53
26.74
f1 (ppm)

Compound 4d

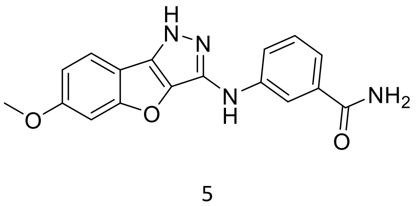

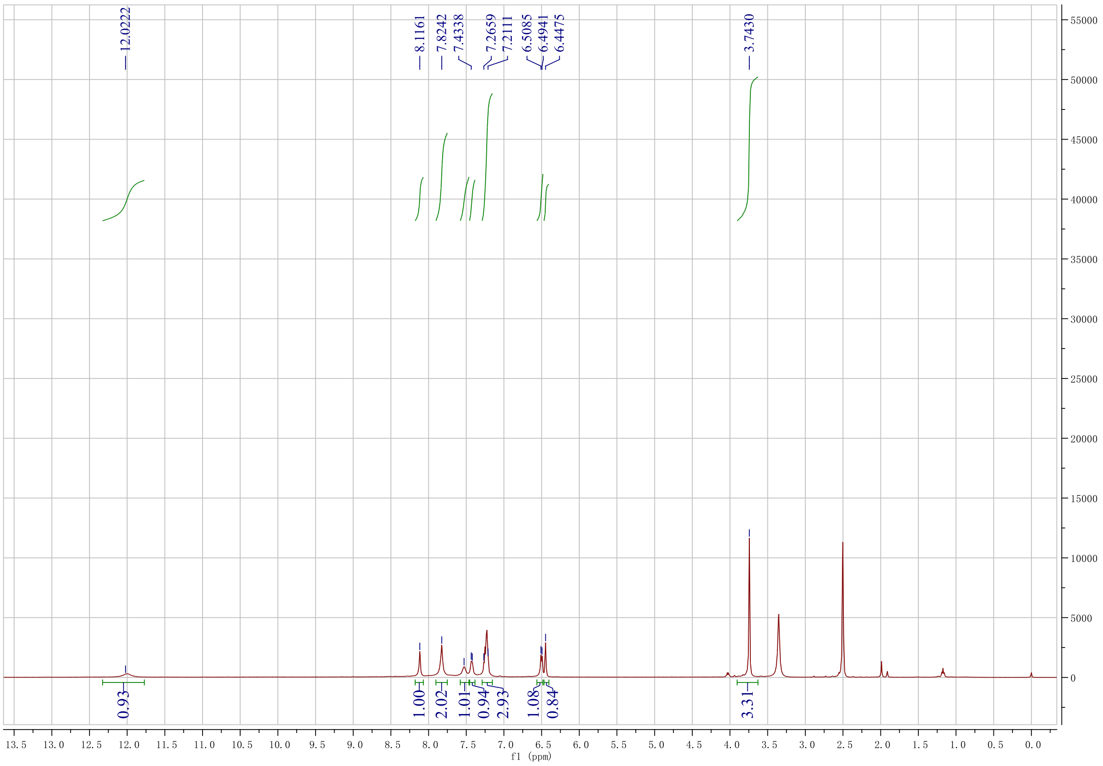
12.0222
8.1161
7.8242
7.4338
7.2659
7.2111
6.5085
6.4941
6.4475
3.7430
0.93
1.00
2.02
1.01
0.94
2.93
1.08
0.84
3.31
f1 (ppm)

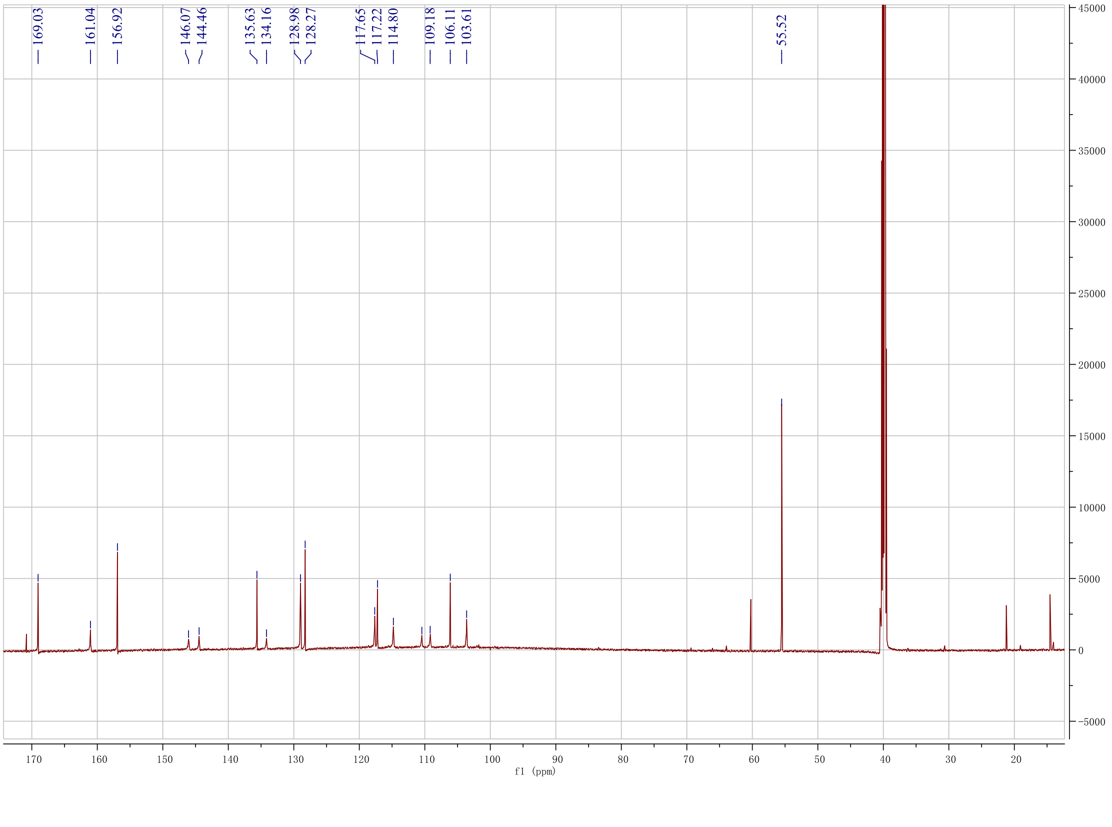
169.03
161.04
156.92
146.07
144.46
135.63
134.16
128.98
128.27
117.65
117.22
114.80
109.18
106.11
103.61
55.52
f1 (ppm)

Compound 4e

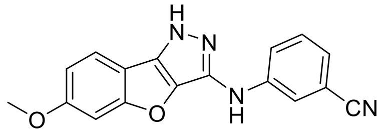
H
N
N
N
H
O
O
CN

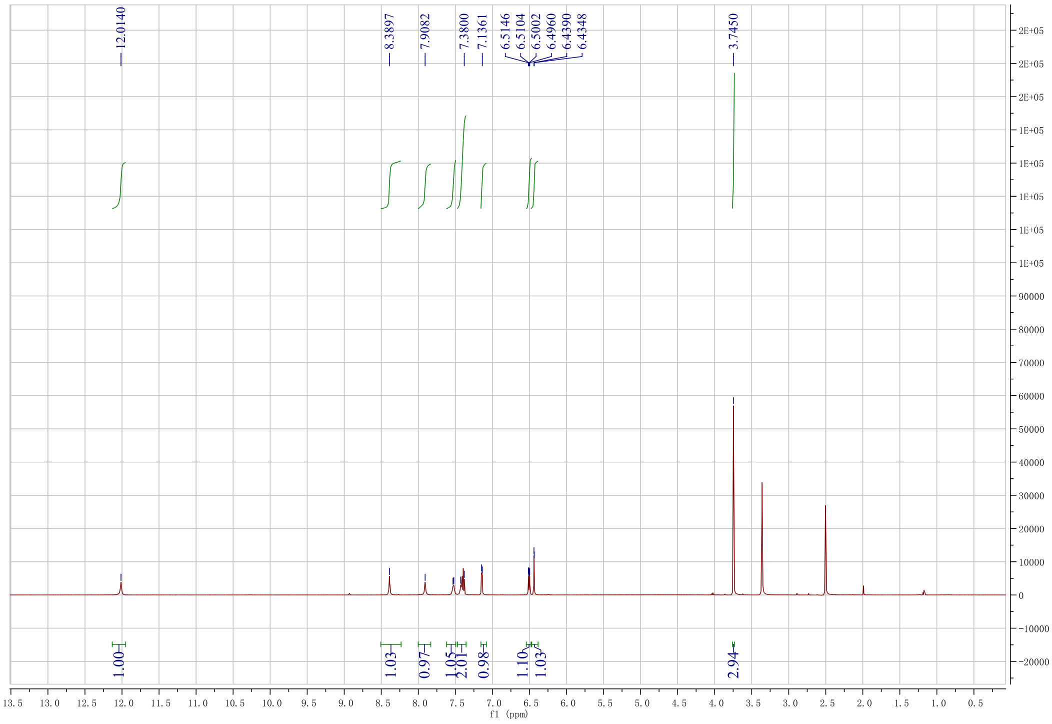
12.0140
8.3897
7.9082
7.3800
7.1361
6.5146
6.5104
6.5002
6.4960
6.4390
6.4348
3.7450
1.00
1.03
0.97
1.05
2.01
0.98
1.10
1.03
2.94
f1 (ppm)

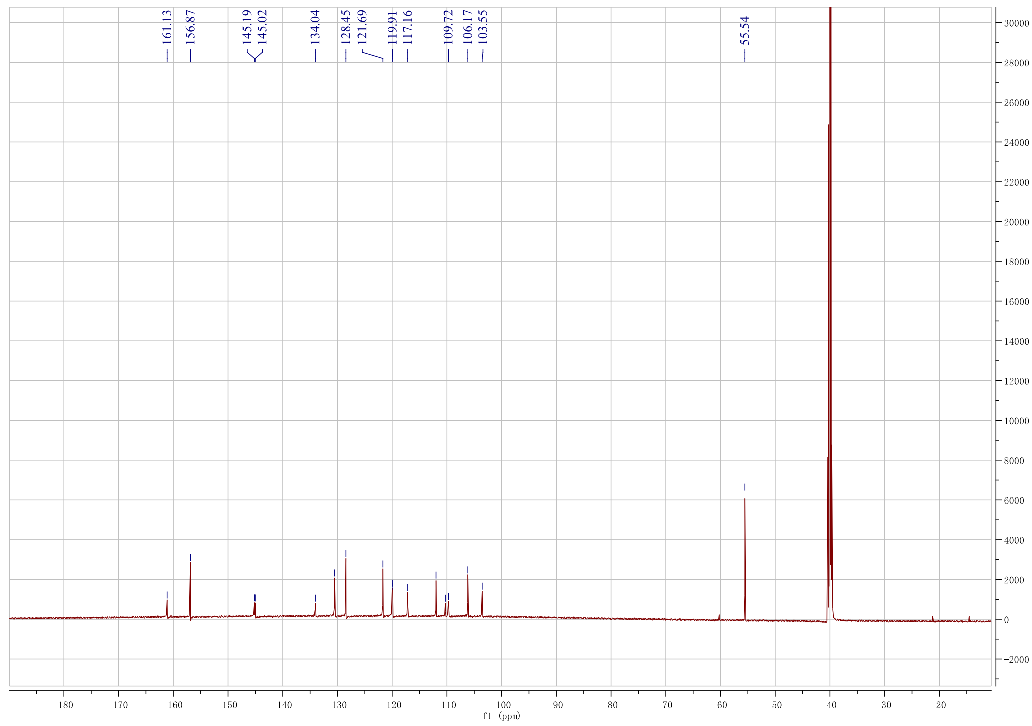
161.13
156.87
145.19
145.02
134.04
128.45
121.69
119.91
117.16
109.72
106.17
103.55
55.54
f1 (ppm)

Compound 5a

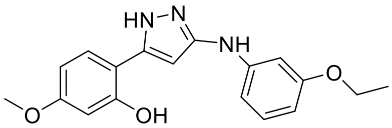
HN
N
NH
O
O
OH

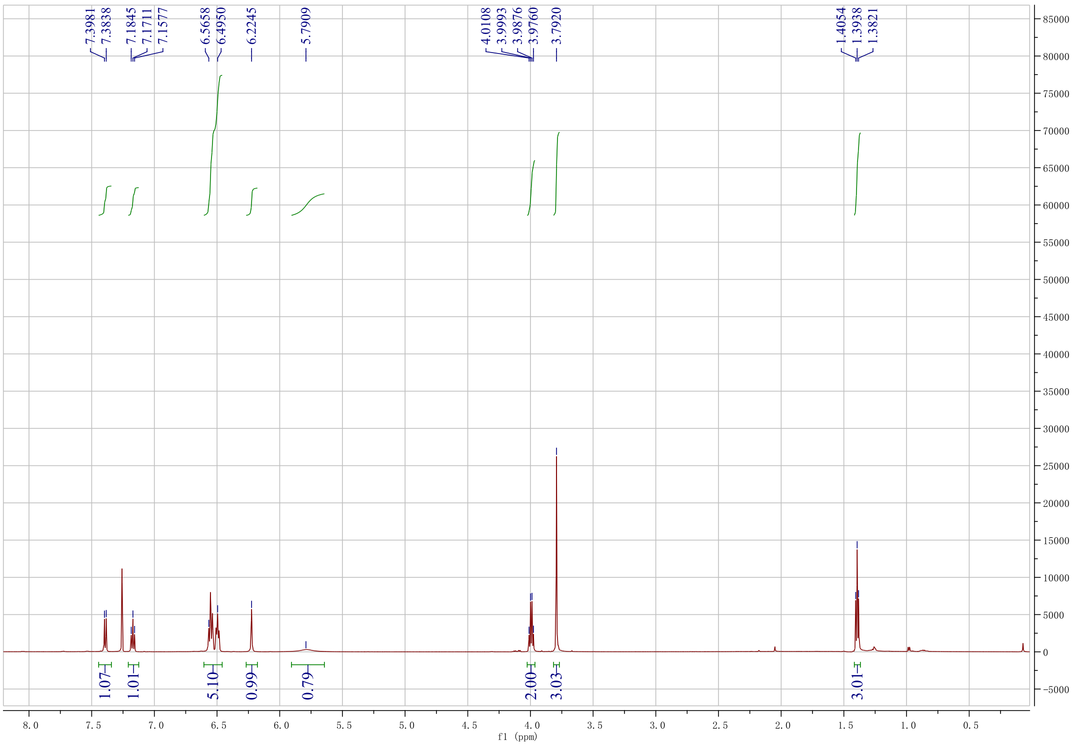
7.3981
7.3838
7.1845
7.1711
7.1577
6.5658
6.4950
6.2245
5.7909
4.0108
3.9993
3.9876
3.9760
3.7920
1.4054
1.3938
1.3821
1.07
1.01
5.10
0.99
0.79
2.00
3.03
3.01
f1 (ppm)

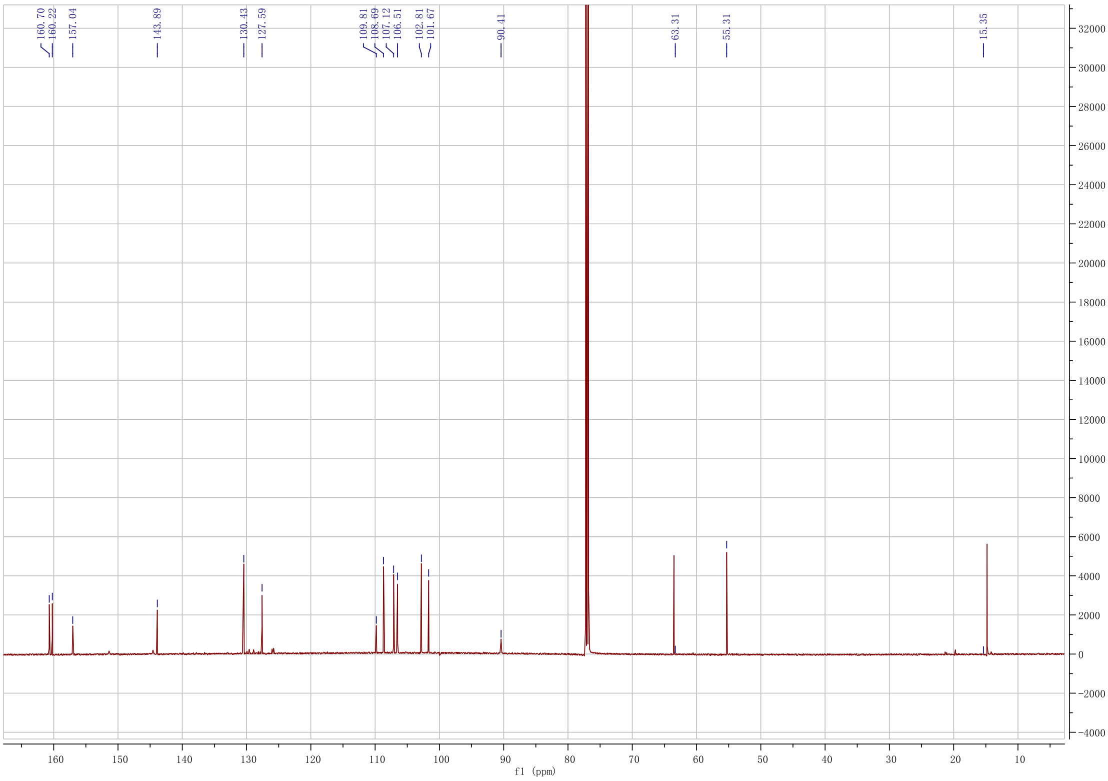
160.70
160.22
157.04
143.89
130.43
127.59
109.81
108.69
107.12
106.51
102.81
101.67
90.41
63.31
55.31
15.35
f1 (ppm)

Compound 5b

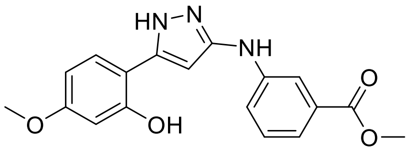
HN
N
NH
O
O
OH
O

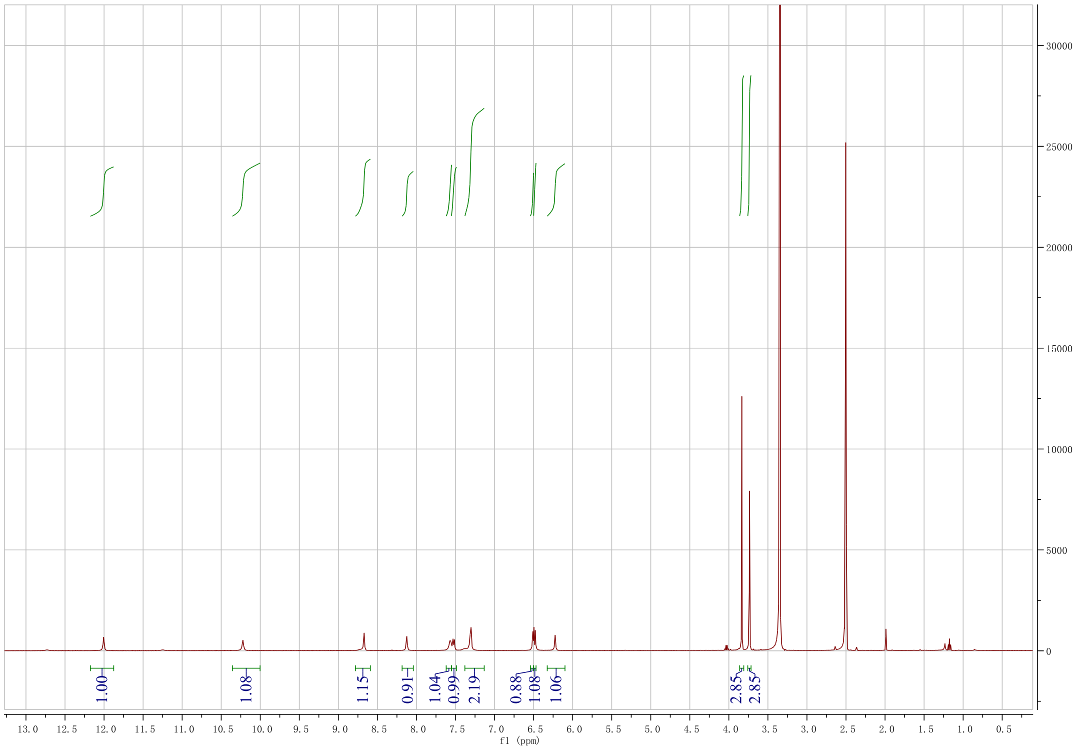
1.00
1.08
1.15
0.91
1.04
0.99
2.19
0.88
1.08
1.06
2.85
2.85
f1 (ppm)

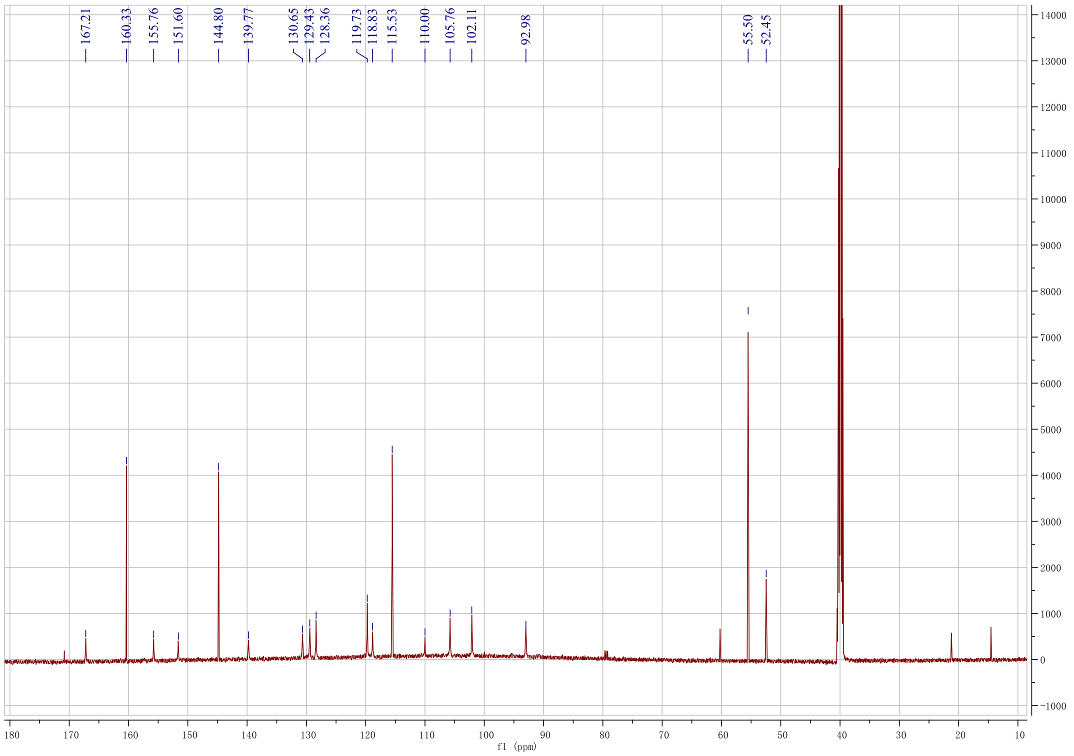
167.21
160.33
155.76
151.60
144.80
139.77
130.65
129.43
128.36
119.73
118.83
115.53
110.00
105.76
102.11
92.98
55.50
52.45
f1 (ppm)

Compound 5c

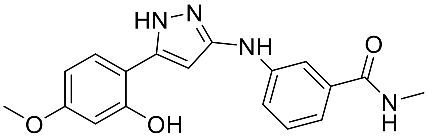
HN
N
NH
O
N
H
O
OH

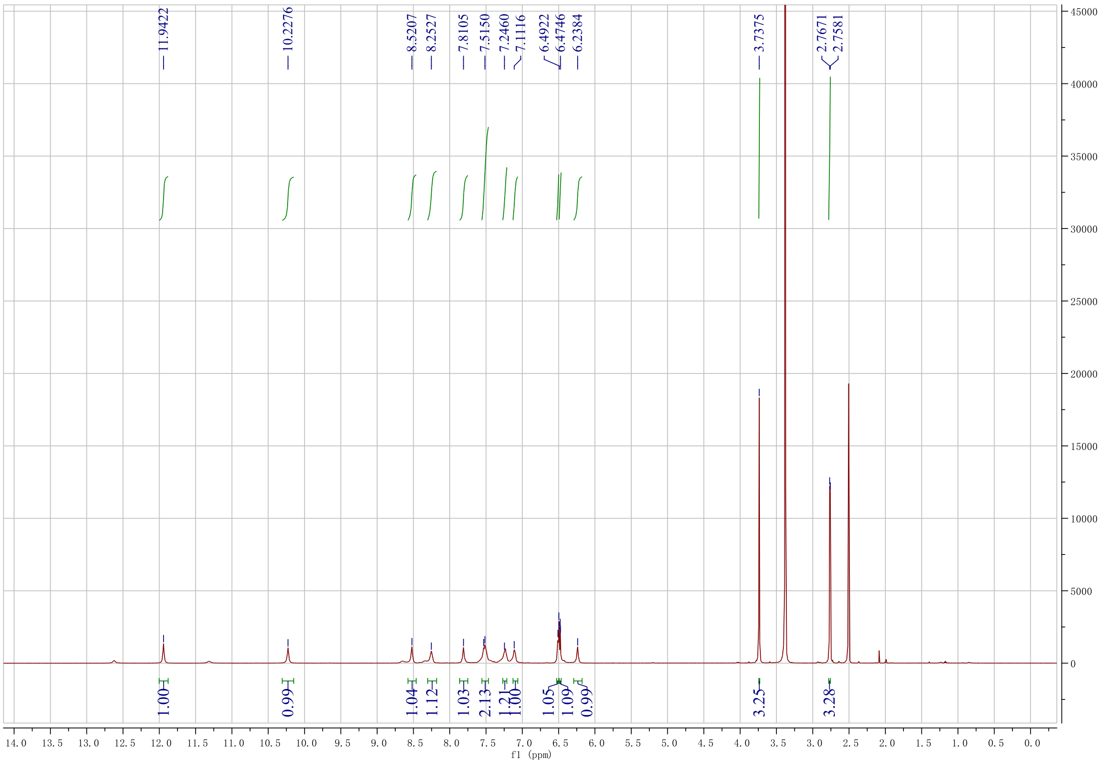
11.9422
10.2276
8.5207
8.2527
7.8105
7.5150
7.2460
7.1116
6.4922
6.4746
6.2384
3.7375
2.7671
2.7581
1.00
0.99
1.04
1.12
1.03
2.13
1.21
1.00
1.05
1.09
0.99
3.25
3.28
f1 (ppm)

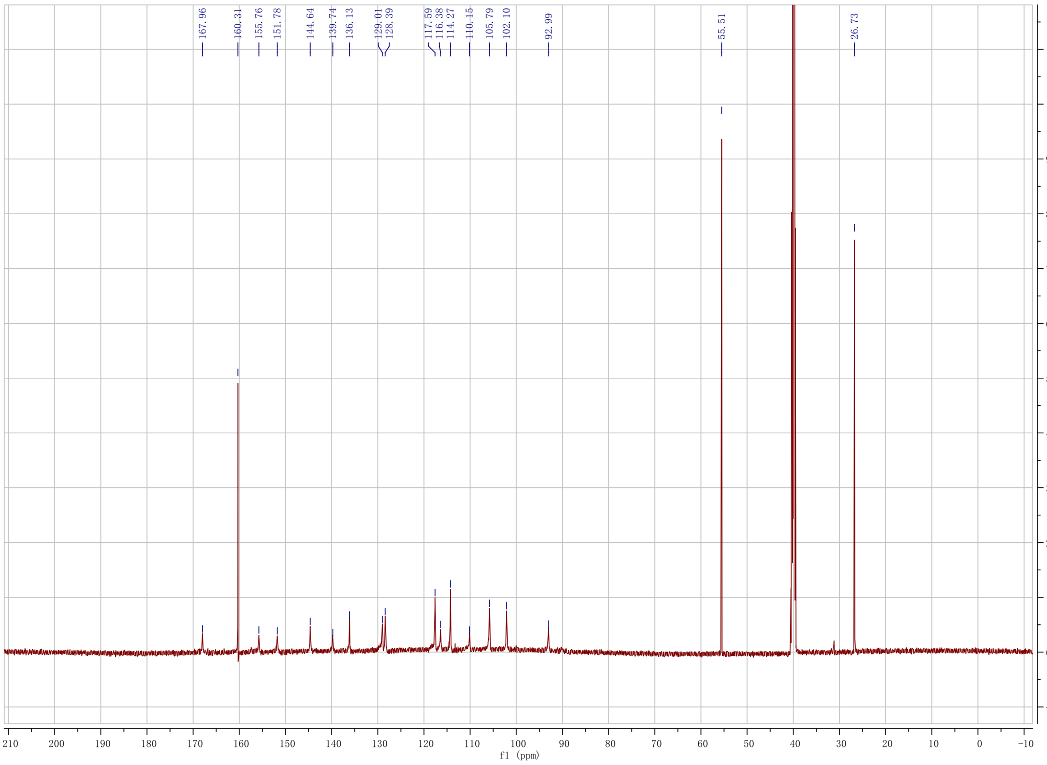
167.96
160.31
155.76
151.78
144.64
139.74
136.13
129.01
128.39
117.59
116.38
114.27
110.45
105.79
102.10
92.99
55.51
26.73
f1 (ppm)

Compound 5d

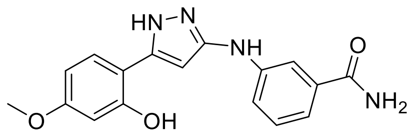
HN
N
NH
O
NH2
O
OH

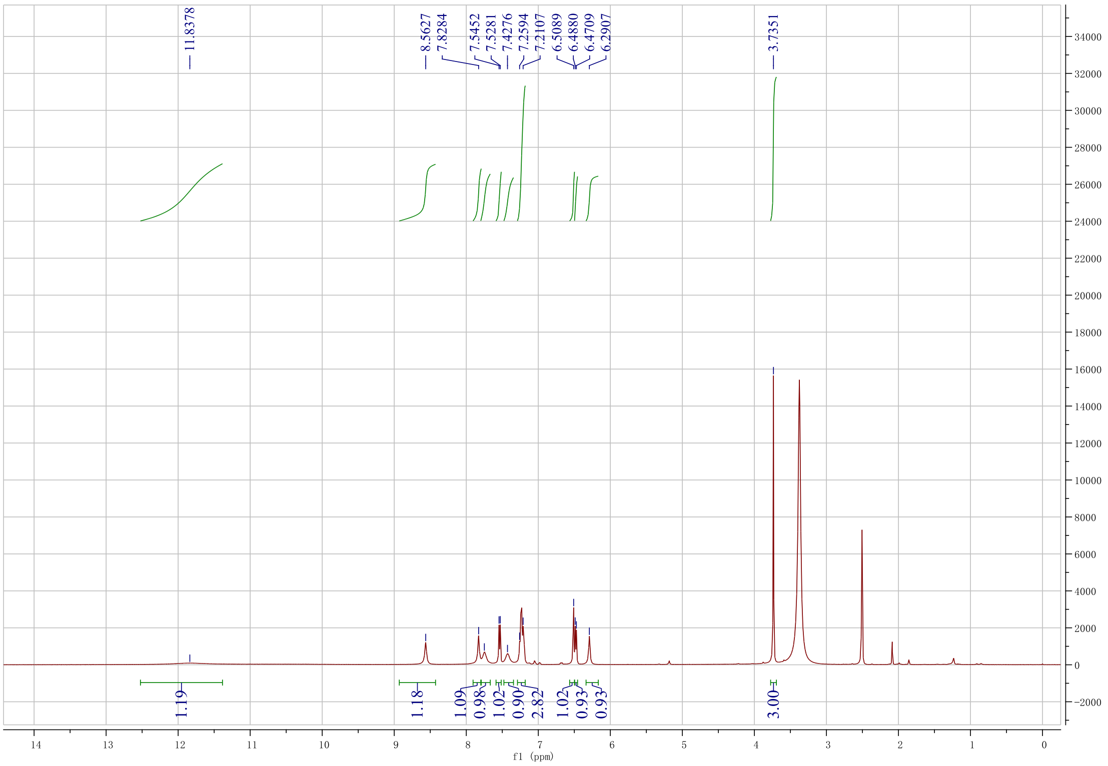
11.8378
8.5627
7.8284
7.5452
7.5281
7.4276
7.2594
7.2107
6.5089
6.4880
6.4709
6.2907
3.7351
1.19
1.18
1.09
0.98
1.02
0.90
2.82
1.02
0.93
0.93
3.00
f1 (ppm)

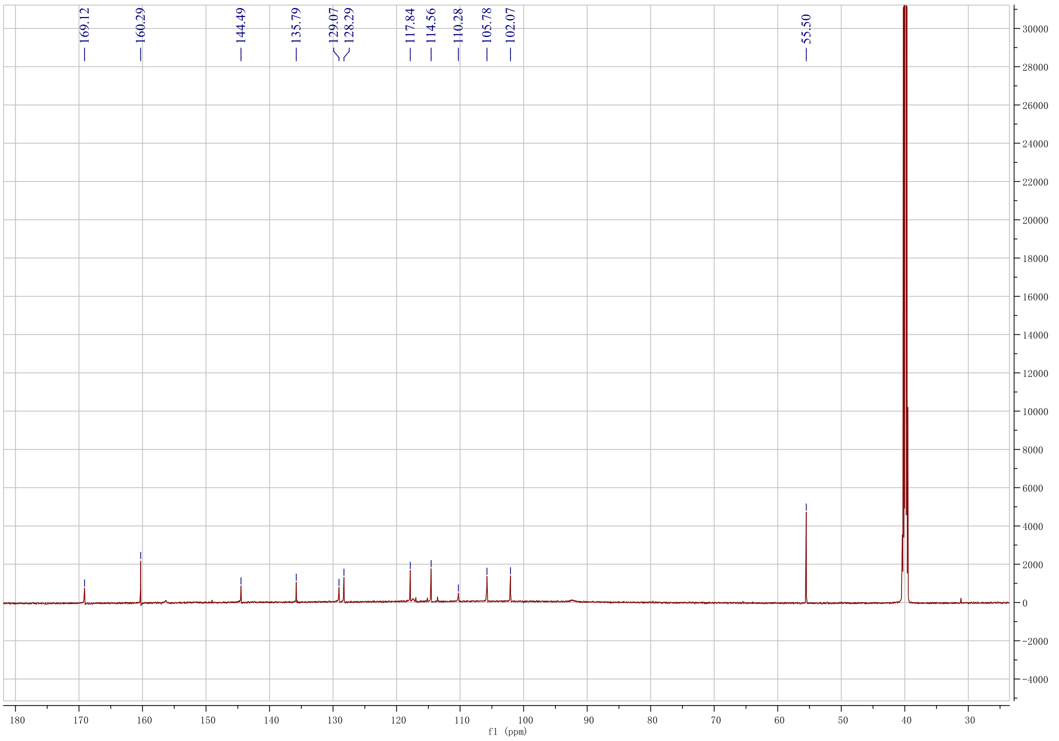
169.12
160.29
144.49
135.79
129.07
128.29
117.84
114.56
110.28
105.78
102.07
55.50
f1 (ppm)

Compound 5e

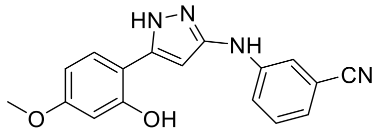
HN
N
NH
CN
O
OH

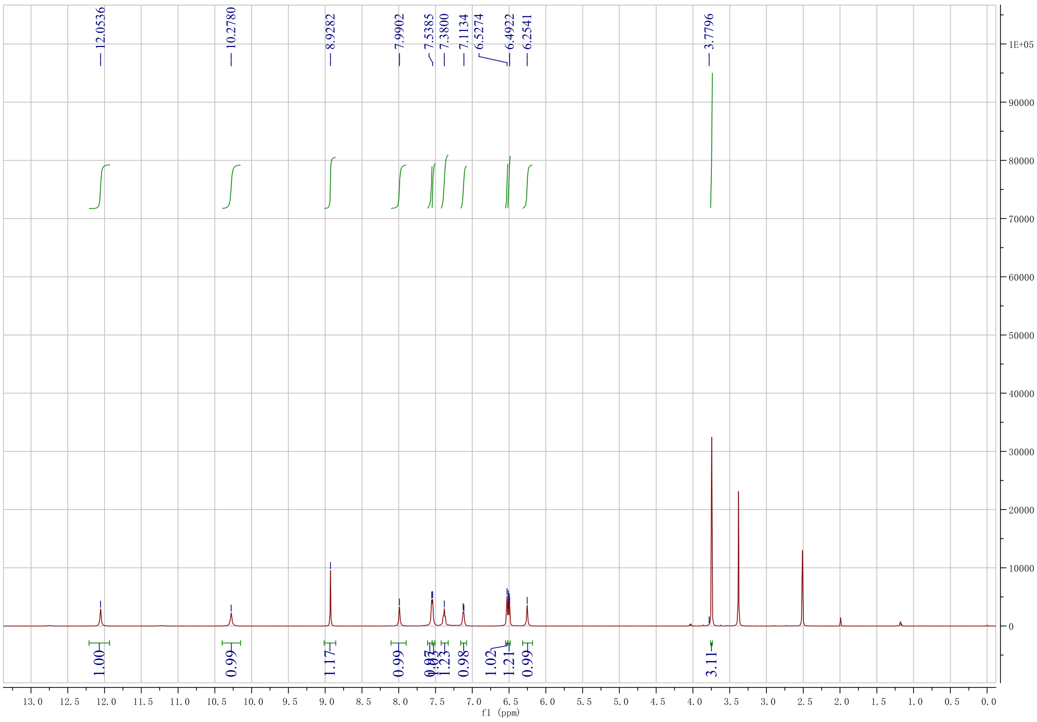
12.0536
10.2780
8.9282
7.9902
7.5385
7.3800
7.1134
6.5274
6.4922
6.2541
3.7796
1.00
0.99
1.17
0.99
1.23
0.98
1.02
1.21
0.99
3.11
f1 (ppm)

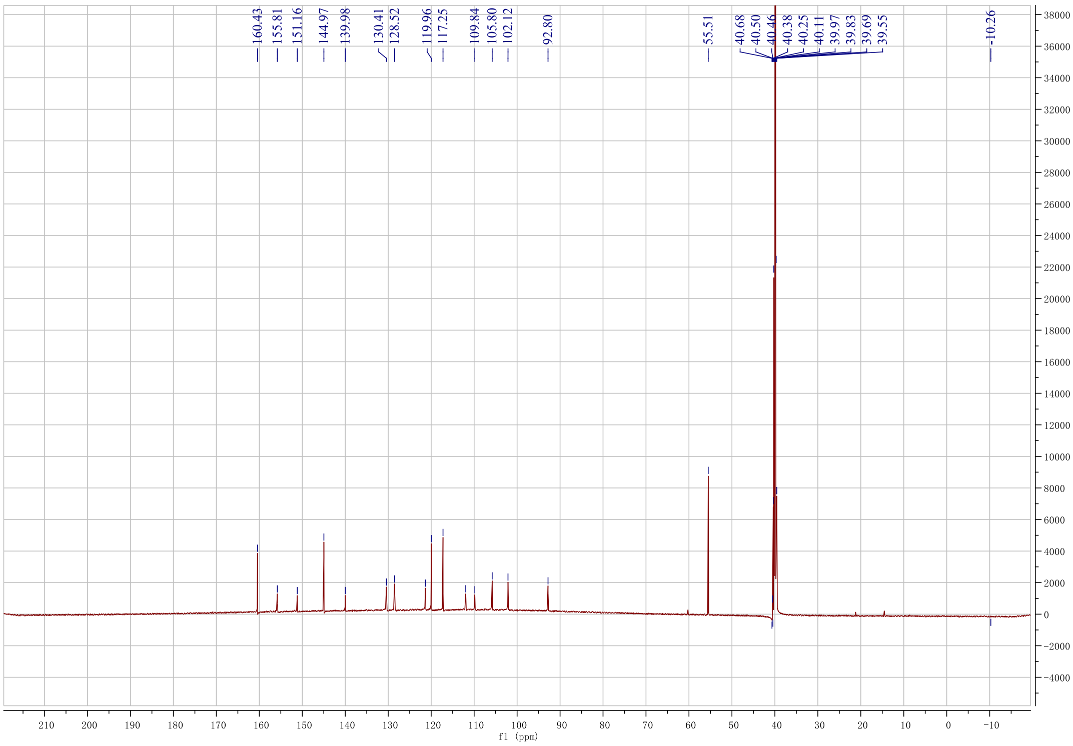
160.43
155.81
151.16
144.97
139.98
130.41
128.52
119.96
117.25
109.84
105.80
102.12
92.80
55.51
40.68
40.50
40.46
40.38
40.25
40.11
39.97
39.83
39.69
39.55
-10.26
f1 (ppm)
